# Toll-like receptor 2 activation and comedogenesis: implications for the pathogenesis of acne

**DOI:** 10.1186/1471-5945-13-10

**Published:** 2013-09-06

**Authors:** Joanne Louise Selway, Tomasz Kurczab, Terence Kealey, Kenneth Langlands

**Affiliations:** 1 The Clore Laboratory, University of Buckingham, Hunter Street, Buckingham MK18 1EG, UK

**Keywords:** Acne vulgaris, Toll-like receptors, Peptidoglycan, Interleukin-1alpha, Keratinocytes

## Abstract

**Background:**

Acne is a common disorder of the human pilosebaceous unit, yet the mechanisms underlying hyperkeratinisation and subsequent inflammation (comedogenesis) remain to be determined, although cutaneous pathogens are implicated. Previously, it was reported that the release of the cytokine interleukin-1α (IL-1α) by keratinocytes of the sebaceous duct was pivotal in the life cycle of the comedone, mediating both its development and its spontaneous resolution. Toll-like receptors are a family of molecules that recognise pathogen associated molecular patterns (PAMPs) presented by microorganisms, initiating a signalling cascade terminating in the release of antimicrobial compounds and cytokines.

**Methods:**

We used *ex vivo* sebaceous gland and primary monolayer keratinocyte culture, alongside ELISAs, immunohistochemistry, Western blotting and RT-PCR to investigate the contribution of TLR activation to acne pathogenesis.

**Results:**

We found TLR2 to be expressed in basal and infundibular keratinocytes, and sebaceous glands, and its activation provoked the release of IL-1α from primary human keratinocytes *in vitro*. The exposure of microdissected human sebaceous glands to PAMPs specific for TLR2 *in vitro* resulted in a pattern of IL-1α like cornification after seven days of exposure.

**Conclusions:**

TLR activation and secretion of IL-1α from keratinocytes may be initiating steps in comedogenesis and, therefore, critical to the pathophysiology of acne.

## Background

Acne is characterized by the formation of comedones, but the etiology of these lesions remains unclear, although cutaneous pathogens are most likely involved. High levels of the pro-inflammatory cytokine IL-1α were reported in acne lesions *in vivo*[1], and previous work revealed that exposure of isolated infundibula and pilosebaceous units to IL-1α *in vitro* induced comedone formation [2,3]*.* Because comedone formation reflects the development of infundibular epidermal scaling, this finding is important because it suggests that IL-1α, whose role in the epidermis remains to be fully elucidated, is a mediator of scaling in inflammatory skin disease. We sought to establish a link between cutaneous flora, the release of IL-1α and the acne life-cycle.

The epidermis provides the first line of defense against invasion by pathogens. Whilst the epidermis contains antigen-presenting Langerhans cells of the adaptive immune system [4], keratinocytes also have inherent mechanisms to combat infection. This innate immunity is a remnant of an ancient host defense mechanism shared with lower organisms, pre-dating adaptive immunity. The activation of the prototypical innate Drosophila receptor Toll is central to this innate response, leading to the release of antifungal factors [5]. A related family of Toll-like receptors (TLRs) are widely expressed in mammalian tissues, particularly epithelia, and growing body of research indicates the importance of innate immunity in cutaneous pathology [6-9]. Eleven mammalian Toll homologues have been characterised to date, each recognizing a discrete set of invariant moieties associated with infectious agents called pathogen associated molecular patterns, or PAMPs [10]. Notable PAMPs include peptidoglycan (PGN) a component of the coats of gram-positive bacteria (including *P. acnes*), lipopolysaccharide (LPS) from gram-negative bacteria, or unmethylated CpG DNA characteristic of viruses, which activate TLR2, TLR4 and TLR9 respectively [reviewed in [11]]. PAMPs are recognised by a leucine-rich extracellular TLR domain, initiating a signal transduction cascade via an intracellular interleukin-1 receptor (IL-1R)-like region, characteristically leading to the release of antibacterial compounds (β-defensins and reactive oxygen species) and cytokines (including IL-1α) via an NFκB-dependent mechanism [10,11].

There is much speculation regarding the source of infundibular IL-1α in acne, and there are reports of release either by keratinocytes [3,12,13] or by the cells of the immune system [14,15]. Since IL-1α release is a consequence of TLR signaling in many cell types including keratinocytes [10,13], we wished to investigated if the exposure of sebaceous glands maintained *ex vivo to* PAMPs induced hypercornification via the TLR-stimulated release of IL-1α.

## Methods

All materials were obtained from Sigma-Aldrich Ltd. (Poole, UK), unless otherwise stated.

### Tissue

Normal human mid-line chest skin was obtained from patients aged 48 to 76 yr undergoing cardiothoracic surgery at Papworth Hospital (Huntington, UK). Sebaceous glands were microdissected as previously described [16]. Both University of Buckingham School of Science and Medicine Ethical Committee approval and informed patient consent were obtained. Tissues were either formalin fixed and paraffin embedded for subsequent histological evaluation, or used immediately for organ culture.

### Organ and tissue culture

Normal human epidermal keratinocytes (NHEK, Invitrogen, Paisley, UK) were maintained in keratinocyte serum free medium (Invitrogen, Paisley, UK) as standard. Sebaceous glands were maintained *ex vivo* on gelatine sponges (Gelfoam, Upjohn, Kalamazoo, MI, USA) at 37°C in a humidified 5% CO2: 95% air environment for 7 days in William’s E medium (without phenol red), supplemented with 2 mM L-glutamine, 500 ng/ml hydrocortisone, sodium selenite (20 μM), triiodothyronine (10 nM), 1% trace element mix (all supplements from Invitrogen), 11.1 mM glucose, 10 μg/ml bovine pituitary extract, 100 U/ml penicillin, 100 μg/ml streptomycin and 2.5 μg/ml amphotericin B. TLR agonists used in the organ culture experiments were LPS (Cat. No. L8643)*,* PGN *(*Cat. No. 77140), and LTA (Cat. No. L2515)*.* Sebaceous glands were incubated in the presence or absence of IL-1α (1 ng/μl), or relevant PAMPs (25 μg/ml). Antibodies raised against human TLR2 and TLR4 were obtained from HyCult Biotechnology bv (Uden, Netherlands) and used at 100 μg/ml to block the effect of the PAMPs. Organ culture experiments consisted of at least 3 biological replicates per treatment group, with at least three glands studied from each donor.

### RT-PCR

RNA was prepared from whole sebaceous glands or cultured cells with TRI-reagent, and 1 μg total RNA was used in first strand cDNA synthesis with random hexamers (Pharmacia) according to the manufacturers’ recommendations*.* 35 cycles of PCR (each of 94°C, 55°C and 72°C for 1 min) were performed as standard using specific primers for TLR2 (TLR2_FOR- GATGCCTACTGGGTGGACAA; TLR2_REV- TTGACAGCTCAGGGATGTTG) (MWG-Biotech, Ebersberg, Germany).

### Immunohistochemistry

4 μm paraffin sections of chest skin derived from 10 different male donors were dewaxed in Histoclear (National diagnostics, Hull, UK) and rehydrated in graded ethanols prior to incubation with an anti-TLR2 goat polyclonal antibody at 37°C overnight at (sc-8690 used at 20 μg/ml, Santa Cruz, La Jolla, USA). Specificity was evaluated by co-incubating primary antisera with a five-fold excess of a Santa Cruz blocking peptide (sc-8690 P, Santa Cruz, La Jolla, USA). Following incubation with an appropriate HRP-conjugated secondary antiserum for 45 minutes at room temperature, slides were developed with ImmPACT DAB peroxidase substrate (Vector Laboratories, Peterborough, UK) according to the manufacturer’s recommendations and counterstained with haematoxylin before clearing in Histoclear and mounting in VectaMount hardset mountant (Vector Laboratories, Peterborough, UK). Tissue sections were stained in triplicate and samples from different individuals were stained contemporaneously to facilitate comparison.

### IL-1α ELISA assays

IL-1α levels in 100 μl medium collected after 2 hr treatment of pre-confluent NHEK with PGN (10 μg/ml) was assayed by ELISA (MESOScale Discovery, Gaithersburg, MD) according to the manufacturer’s recommendations. In order to assess specificity, NHEK incubated with PGN and a ten-fold excess (by mass) of a TLR2 neutralising antibody were included in the study. All assays were performed in triplicate.

### Western blotting

Pre-confluent primary keratinocytes were homogenised in RIPA buffer (150 mM NaCl, 10 mM Tris-Cl pH7.2, 5 mM EDTA, 0.1% SDS, 1.0% Triton-X100, 1.0% sodium deoxcycholate, 1 mM phenylmethylsulphonylfluoride). 20 μg protein was electrophoresed through a 4-12% pre-cast bis-tris NuPage mini-gel (Invitrogen) and transferred to PVDF membranes (Millipore) by wet electroblotting as standard. Immunodetection was performed using an anti-IκB polyclonal antibody (used at 1:1000 dilution, Santa Cruz) and chemiluminescent detection (WesternBreeze, Invitrogen) as recommended.

## Results

### TLR2 is expressed in human sebaceous glands and keratinocytes

**Figure 1:**
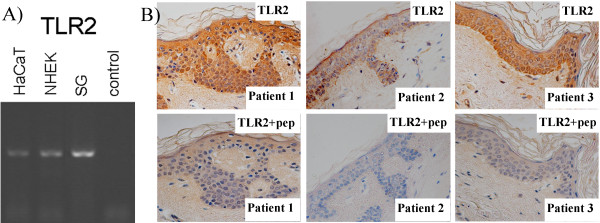
**TLR Expression *in vitro *and *in vivo*. A)** TLR2 expression was assessed by RT-PCR in cDNA prepared from HaCaT keratinocytes (first lane), NHEK (second lane) or freshly-isolated human sebaceous glands (third lane, representative of ten different individuals). A no-template cDNA control was included (fourth lane). **B)** TLR2 antisera were used to evaluate receptor localisation in skin isolated from ten individuals and representative expression patterns are shown, with and without a blocking peptide (pep). Images were captured at 10 × original magnification.

Expression of TLR2 mRNA in sebaceous glands from ten individuals, as well as in normal human epidermal keratinocytes (NHEK) and HaCaT keratinocytes, was profiled by PCR (Figure 1A). Qualitatively, expression of TLR2 was consistently found to be higher in primary sebaceous gland tissue compared to isolated keratinocytes.

### TLR expression characterises the human infundibulum and basal interfollicular keratinocytes *in situ*

TLR2 immunoreactivity was evaluated in tissue isolated from ten individuals, and representative patterns are shown in Figure 1B. In the interfollicular epidermis, expression was consistently most abundant in the basal layer, and this predominantly basal pattern of immunolocalisation was also observed in infundibular keratinocytes. The specificity of immunoreactivity was confirmed by co-incubation with a specific TLR2 antigen blocking peptide, following which no staining was observed (Figure 1B).

### IL-1α is released in response to TLR2 activation

**Figure 2:**
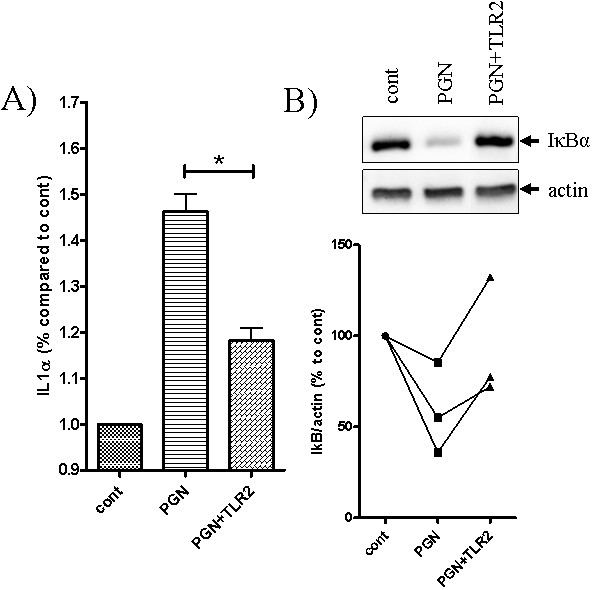
**TLR activation results in IL-1α release and NF-κB activation. A)** Preconfluent NHEK were treated with PGN in the presence or absence of a TLR2 neutralising antibody, and levels of IL-1α release determined by ELISA. **B)** Three separate cultures of preconfluent NHEK were harvested following 90 min exposure to PGN, and IκB expression relative to β-actin was determined by Western blotting.

TLR2 activation with PGN provoked the rapid release of IL-1α from NHEKs (Figure 2A). PGN stimulated a 46% increase in IL-1α secretion relative to vehicle only controls (p < 0.05). Moreover, the effect was specific as the co-incubation of PGN with a TLR2 neutralising antibody resulted in a significant reduction in IL-1α levels in the media.

### PAMP-mediated TLR2 stimulation in NHEKs activates the NFкB pathway

Western blotting was used to determine the degradation of the NFκB repressor IκB in response to PAMP stimulation of NHEK. A reduction in IκB signal in response to PGN treatment was detected after two hours of PAMP incubation, an effect that was inhibited in the presence of the TLR2 blocking antibody (Figure 2B).

### PAMPs cause hypercornification in sebaceous glands maintained *ex vivo*

**Figure 3:**
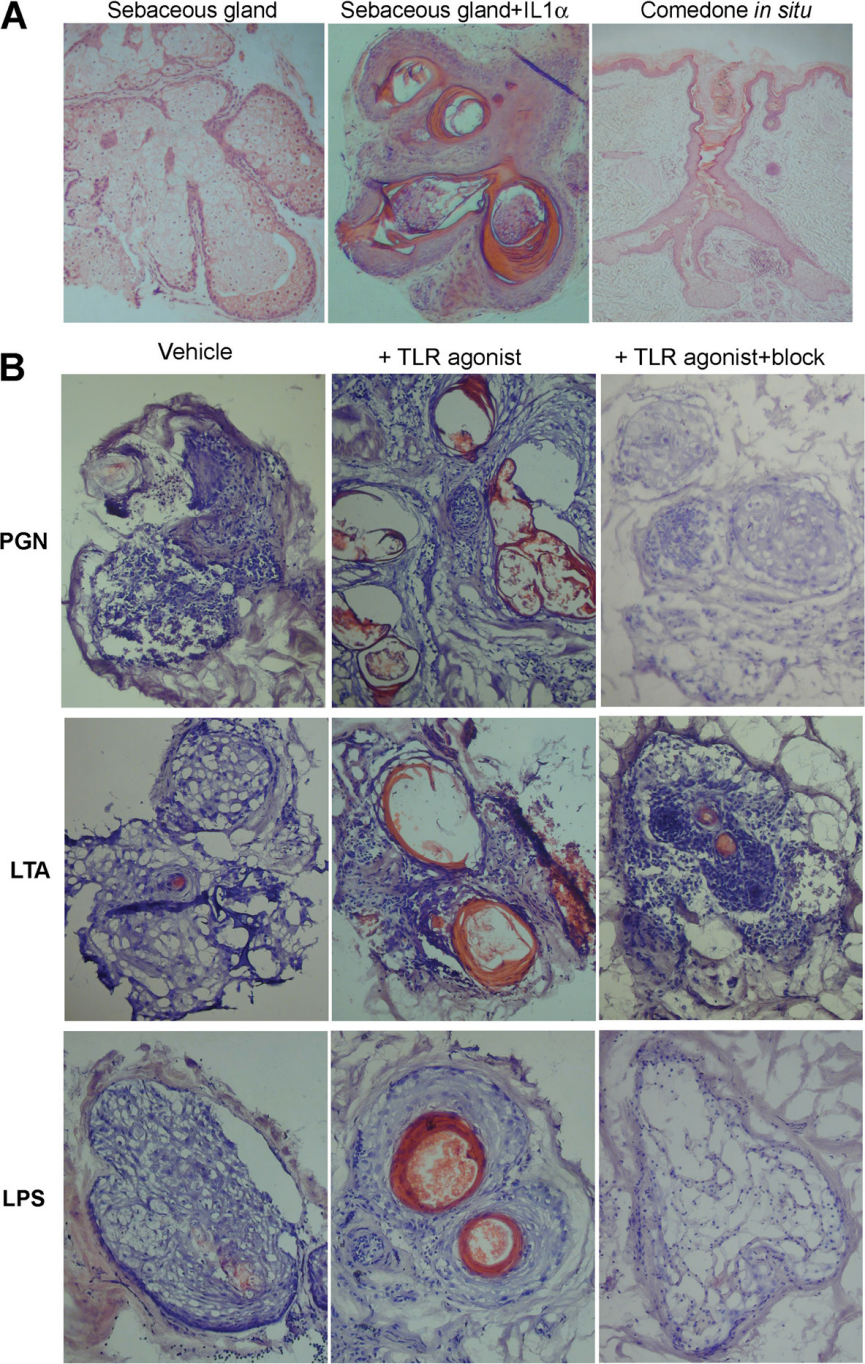
**TLR activation promotes hypercornification in isolated human sebaceous glands maintained *ex vivo*. A)** Histological sections of freshly-isolated and IL-1α treated sebaceous glands are shown, along with an example of a comedone *in situ*. **B)** Morphology representative of 3 experiments showing sebaceous glands treated with vehicle only (control), TLR2 (LTA and PGN) or TLR4 agonists (LPS), or TLR agonists pre-incubated with a blocking antibody (block). All images are shown at 10 × original magnification.

The normal lobular structure of a freshly-isolated sebaceous gland is shown in Figure 3A, along with the cornifying effects of 1 ng/μl IL-1α treatment for seven days. A section of a comedone *in situ* is also shown. Microdissected sebaceous glands were maintained in the presence or absence of different TLR agonists (LTA, PGN and LPS) for seven days prior to histological analysis (Figure 3B) after which time hypercornification (characteristic of acne lesions *in vivo*) was assessed. Whilst there was some loss of structure in those organs maintained for seven days in the presence of vehicle alone, increased cornification was not detected. Exposure to both PGN and LTA treatment resulted in pattern of cornification similar to that observed following IL-1α treatment, which was blocked following co-incubation of PAMPs with a fourfold excess (by mass) of a TLR2 neutralising antibody.

The skin is also colonised by gram-negative commensals such as *P. aeruginosa* (which act via TLR4 activation) and the exposure of isolated glands to LPS also specifically-increased cornification, which was blocked following co-incubation with an excess of a TLR4 neutralising antibody. All images are representative of three different experiments.

## Discussion

The expression of functional TLRs in both interfollicular and infundibular human keratinocytes is consistent with an innate role for these cells in the sensing of, and response to, bacterial pathogens [17]. The majority of cutaneous pathogens are gram-positive and, therefore, likely to initiate a response via TLR2. As *P. acnes* exposure leads to increases in TLR2 (as well as TLR4) expression in NHEK, and TLR2 expression was reported within acne lesions *in situ*[18]*,* we choose to further investigate the role of this receptor in keratinocyte and sebaceous gland function.

Although keratinocytes are known to express TLR2, the precise compartmental localization of this receptor in the epidermis is not clear [18-22]. Our observation of predominantly basal localization of TLR2 in a range of human skin samples agrees with the reports of both Baker *et al.*[19] and Curry *et al.*[20], but some variation between individuals, or indeed studies, would be expected (as reported by Pivarsci *et al.*[21] and Jugeau *et al.*[18]) as these receptors are readily induced, notwithstanding any genetic differences [23].

Viable stationary-phase *P. acnes* directly-activates NHEK via TLR2, stimulating IL-1α release [12]. As the treatment of NHEK with LTA and PGN was reported to trigger NFκB activation via TLR2, in turn regulating the release of the potent chemokine IL-8 [17,21], it is rational to investigate the role of PAMPs in the pathogenesis of acne. We found that the treatment of primary cultures of keratinocytes with TLR2 agonists provoked the release of IL-1α, confirming our hypothesis and previously published results [17,21]. This is likely to be a post-translational event as previous observations indicated that stimulation with micro-organisms (including *P. acnes*) was unable to stimulate the synthesis of IL-1α in keratinocytes [24,25]. In addition, in our organ-maintained pilosebaceous units, we were able to show that PAMP treatment led to a pattern of hypercornification similar to that created by IL-1α exposure (and, indeed, that seen in comedones *in situ*), via both TLR2- and TLR4-dependent mechanisms. Furthermore, normal morphology was maintained when PAMP activity was neutralized with specific TLR blocking antibodies. This suggests that PAMPs mediate their effects on sebaceous glands via TLRs. The presence of a TLR2-dependent innate immune response in sebocytes was previously documented [25,26], but the role of IL-1α, in addition to that of lipid mediators such as free fatty acids, is complex.

We recognize that the removal of organs from their natural environment in our *ex vivo* model does not allow us to investigate the contribution of the immune system. This is an important consideration as the response triggered by skin resident immune cells could modulate the effect of PAMPs through both TLR-dependent and TLR-independent mechanisms. That we were able to provoke a response in normal human skin (or at least skin without frank evidence of acne), which is associated with a low level of infiltrating immune cells, suggest that our model is able to recapitulate comedogenesis, at least in part. One could also investigate the role of TLR agonism in human sebocyte monolayer culture, or in sebaceous glands isolated from individuals with acne, but we feel that our model provides a good compromise between biological relevance and feasibility.

The role of pro-inflammatory cytokines in enhanced lipogenesis and acne pathogenesis is complex [24,25,27-32]. Increased sebum levels in adolescents, perhaps exacerbated by elevated free fatty acid availability resulting from exposure to Western diets [30], may promote cutaneous *P. acnes* colonization [32]. Furthermore, other hormonal mediators may contribute to a ‘perfect storm’ of acne susceptibility. For example, elevated IGF1 signaling can suppress the transcription of anti-microbial peptides [33], thereby priming the innate immune response. Thus, enhanced sebum levels, high IGF1 signaling and free fatty acid availability could act in concert to create a pro-inflammatory state primed for comedogenesis [28]. Prominent in this pro-inflammatory state is IL-1α [17,21], as well as other cytokines such as TNFα [29,30].

## Conclusions

**Figure 4:**
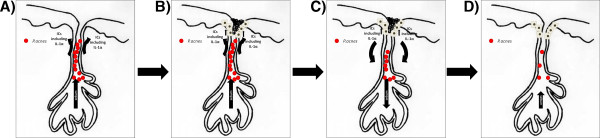
**A model of TLR activation and the life-cycle of acne. A)***P. acnes* colonises the sebaceous gland and stimulates infundibular keratinocytes to release inflammatory cytokines (ICs) including IL-1α via TLR activation. **B)** ICs, including IL-1α, stimulate hypercornification, thus formation of the comedone. **C)** IC secretion, including IL-1α, is also associated with a reduction in lipogenesis in basal sebocytes, thereby reducing the sebum levels and starving the *P. acnes* of nutrients. **D)** Reduction in *P. acnes* levels reduces IC secretion, resolution of the comedone and restoration of sebocyte maturation. Adapted from Downie MM *et al.*[3].

We suggest that IL-1α release from infundibular keratinocyte in response to *P. acnes-* mediated TLR activation is an important step in the complex natural history of the acne lesion (Figure 4). Moreover, IL-1α may contribute to both the creation of a comedogenic cytokine millieu, as well as promoting the eventual sebocyte hypercornification characteristic of acne lesions.

## Competing interests

The authors declare that they have no competing interests

## Authors’ contributions

JLS carried out the immunohistochemistry, ELISA assays and Western blotting, T. Kurczab carried out the RT-PCR, T. Kurczab and KL performed the sebaceous gland dissection, culture and analysis. KL and T. Kealey devised the project and study design. JLS and KL drafted and revised the manuscript. All authors read and approved the final manuscript.

## Pre-publication history

The pre-publication history for this paper can be accessed here:

http://www.biomedcentral.com/1471-5945/13/10/prepub
